# Insights into the Role of VPS39 and Its Interaction with CP204L and A137R in ASFV Infection

**DOI:** 10.3390/v16091478

**Published:** 2024-09-17

**Authors:** Katarzyna Magdalena Dolata, Axel Karger

**Affiliations:** Institute of Molecular Virology and Cell Biology, Friedrich-Loeffler-Institut, Federal Research Institute for Animal Health, Südufer 10, 17493 Greifswald-Insel Riems, Germany

**Keywords:** african swine fever virus, VPS39, CP204L, A137R, protein–protein interaction, virus–host interaction

## Abstract

The African swine fever virus (ASFV) is a large and complex DNA virus that causes a highly lethal disease in swine, for which no antiviral drugs or vaccines are currently available. Studying viral–host protein–protein interactions advances our understanding of the molecular mechanisms underlying viral replication and pathogenesis and can facilitate the discovery of antiviral therapeutics. In this study, we employed affinity tagging and purification mass spectrometry to characterize the interactome of VPS39, an important cellular factor during the early phase of ASFV replication. The interaction network of VPS39 revealed associations with mitochondrial proteins involved in membrane contact sites formation and cellular respiration. We show that the ASFV proteins CP204L and A137R target VPS39 by interacting with its clathrin heavy-chain functional domain. Furthermore, we elaborate on the potential mechanisms by which VPS39 may contribute to ASFV replication and prioritize interactions for further investigation into mitochondrial protein function in the context of ASFV infection.

## 1. Introduction

African swine fever virus (ASFV) is a large double-stranded DNA virus from the *Asfarviridae* family. ASFV causes a highly contagious and deadly disease in domestic swine [1], and the lack of a vaccine or effective treatment results in substantial economic losses for the global pig industry [2].

The ASFV genome varies in size, ranging from 170 to 193 kbp, depending on the virus strain, and encodes 68 structural proteins [3] and more than 100 non-structural proteins [4,5]. Over the past decade, protein–protein interaction (PPI) studies have contributed to identifying certain host cellular factors and processes essential for ASFV replication [6]. Nonetheless, the functions of more than half of the viral genes have yet to be experimentally characterized. Therefore, further progress in identifying ASFV host PPIs is essential for a better understanding of the virus and discovering potential drug targets.

In a previous study, we identified an interaction between the ASFV protein CP204L and the cellular vacuole protein sorting protein VPS39, a homotypic fusion and protein sorting (HOPS) complex component. This interaction modulates the rearrangement of the host endosomal and lysosomal membranes towards the site of virus replication [7]. Furthermore, we showed that CP204L associates with another viral protein, A137R, but we could not confirm their direct interaction.

CP204L, also known as P30 or P32, and A137R proteins, are important for ASFV replication [8,9] and abundantly expressed in infected cells [10]. Additionally, they interact with the host proteins involved in endocytic and autophagy pathways [11,12,13]. Their common characteristics indicate that CP204L and A137R could interact indirectly through a shared interaction partner. This report shows that both ASFV proteins, CP204L and A137R, bind to the host protein VPS39, targeting its specific functional domains.

VPS39 is known to play a role in various aspects of endosomal maturation [14,15], the regulation of cell signaling [16] and autophagy/mitophagy [17], and the formation of membrane contact sites [18]. During SARS-CoV2 infection, VPS39 is sequestered by the viral ORF3a protein, allowing the virus to escape from autophagic lysosomes [19,20]. In the early phase of ASFV infection, VPS39 is an important host factor for viral replication and protein synthesis [7]. However, its precise role in ASFV pathogenesis remains unknown. Therefore, to further elucidate the function of VPS39, we performed a PPI study on ASFV-infected cells.

This brief report identifies novel viral and cellular interactors of VPS39 during ASFV infection, highlighting an enrichment of mitochondrial proteins. We further explore the potential role of VPS39 in mitochondrial–lysosomal crosstalk, energy metabolism, and the functional implications of its interactions with CP204L and A137R within the ASFV infection context.

## 2. Materials and Methods

### 2.1. Cell Culture, Virus Infection, and Transfection

The wild-boar-lung-derived cells (hereafter referred to as WSL) [21], supplied by Friedrich-Loeffler-Institut Biobank (catalog number CCLV-RIE 0379), were cultured in Iscove’s modified Dulbecco’s medium (IMDM) mixed with Ham’s F-12 nutrient mix (1:1 [vol/vol]) supplemented with 10% fetal bovine serum (FBS) at a humidified 37 °C and 5% CO_2_ atmosphere.

The ASFV Armenia/07 isolate was propagated on WSL cells. Passage 20 stocks were generated as previously described [8]. WSL cell monolayers were inoculated with ASFV stock dilutions at an MOI of 2 PFU/cell. Cells were centrifugated for 1 h at 600× *g* and 37 °C. After removal of the inoculum, cells were washed three times with phosphate-buffered saline (PBS), overlaid with fresh medium containing 5% FBS, and incubated at 37 °C with 5% CO_2_. Infection with ASFV was carried out in a biocontainment facility that fulfills the safety requirements for ASF laboratories and animal facilities (Commission Decision 2003/422/EC, chapter VIII).

WSL cells were transiently transfected using K2 transfection reagent (Biontex, Munich, Germany) following the manufacturer’s instructions and harvested 24 h after transfection.

### 2.2. Plasmids

GFP, CP204L-GFP, A137R-GFP, and VPS39-GFP plasmids were previously described [7]. Open reading frame (ORF)-encoding fragments of VPS39 were amplified by PCR (Phusion™ Plus DNA Polymerase; Thermo Scientific™, Darmstadt, Germany) from VPS39-GFP plasmid. Amplification was performed using ORF-specific primers flanked with the Gateway cloning sites 5′-GGGGACAACTTTGTACAAAAAAGTTGGC and 5′-GGGGACAACTTTGTACAAGAAAGTTGG. PCR products were cloned by in vitro recombination into pDONR207 (Gateway System; Invitrogen, Schwerte, Germany). ORF coding sequences were subsequently transferred by the in vitro recombination from pDONR207 into pDEST-EmGFP-vivid (Invitrogen) according to the manufacturer’s recommendations (LR cloning reaction; Invitrogen).

### 2.3. Antibodies

Rabbit antisera specific for ASFV CP204L [22] and A137R (unpublished data) proteins were used at dilutions of 1:20,000 for immunoblotting. The primary antibodies used for immunoblotting included rabbit anti-GFP (Chromotek, Planegg, Germany), anti-FLAG (F1804; Sigma-Aldrich, Taufkirchen, Germany), rabbit anti-VPS39 (PA5-21104; Thermo Fisher, Damstadt, Germany), mouse anti-tubulin (B-5-1-2; Sigma-Aldrich). The secondary antibodies used were peroxidase-conjugated goat anti-mouse and anti-rabbit IgG (Jackson ImmunoResearch, Ely, UK). The additional secondary antibodies used for immunofluorescence were Alexa Fluor 647-conjugated goat anti-rabbit IgG (H + L) and goat anti-mouse IgG (H + L) (Invitrogen).

### 2.4. Immunofluorescence and Microscopy

Coverslips were fixed with 3.7% formaldehyde in PBS for 1 h at room temperature, washed three times for 10 min with PBS, permeabilized with 0.01% Triton X-100 in PBS for 15 min, and then blocked with PBS containing 10% FBS for 1 h. Primary antibodies were incubated for 1 h at room temperature (RT) following three washing steps with PBS and secondary antibody incubation for 1 h at RT. Nuclei were stained with 1 μg/mL Hoechst 33258 (Sigma-Aldrich). Coverslips were then mounted on glass slides using ProLong Glass Antifade Mountant (Invitrogen). Single-slice images were acquired on a Leica DMI6000 TCS SP5 confocal laser scanning microscope (63× objective) and processed with ImageJ software (v.1.52a) [23].

### 2.5. Immunoprecipitation

WSL cells were harvested and lysed on ice in 1 mL cold immunoprecipitation (IP) buffer (50 mM Tris-HCl [pH 7.4], 150 mM NaCl, and 1 mM MgCl_2_ supplemented with Benzonase [25 U/mL; Sigma-Aldrich no. E8263], 0.5% Nonidet P40 substitute [NP-40; Sigma-Aldrich no. I8896], and cOmplete^TM^ mini EDTA-free protease inhibitor cocktail [Roche, no. 04693159001]). Cells were lysed by sonication three times for 30 s each at 80% amplitude (Branson Digital Sonifier 450) and incubated on a tube rotator for 30 min at 4 °C and another 30 min on a thermomixer at 37 °C with constant shaking at 900 rpm. Lysates were cleared by centrifugation at 13,000× *g* and 4 °C for 15 min. Fifty microliters of each lysate were removed for immunoblotting (whole-cell lysate fraction). For each immunoprecipitation reaction, 0.9 mL of cell lysate was incubated with 50 µL GFP-trap or Fab-trap beads (Chromotek, Planegg, Germany) at 4 °C. After 1 h incubation, the beads were washed twice with 1 mL of IP buffer containing 0.05% NP-40, followed by two washes in detergent-free IP buffer. Ten microliters of bead slurry was removed for immunoblotting (IP fraction). The remaining 40 μL beads were kept for mass-spectrometric analysis. Samples were prepared in four independent biological replicates.

### 2.6. Immunoblotting

Beads and whole-cell lysates were boiled in IP buffer, resolved on SDS-PAGE [24] gradient gels (4 to 20% Mini-Protean TGX gels (Bio-Rad, Feldkirchen, Germany), followed by Coomassie Brilliant Blue-G staining [25] or transferred to the nitrocellulose membrane by semidry transfer (Trans-Blot Turbo; Bio-Rad Laboratories) for immunoblot analysis [26]. The membranes were incubated with the indicated specific primary antibodies diluted in Tris-buffered saline with 0.25% Tween 20 (TBST) supplemented with 1% milk. The membranes were washed thrice with TBST and exposed for 1 h to specific horseradish peroxidase-conjugated secondary antibodies. Chemiluminescence detection was performed using the Clarity Western-enhanced chemiluminescence substrate (Bio-Rad), imaged on a C-DiGit blot scanner (LI-COR, Bad Homburg vor der Höhe, Germany), and analyzed with Image Studio software (v.5.2).

### 2.7. Sample Preparation and Mass Spectrometry Analysis

Proteins in lysates and IP fractions were reduced by adding DTT to a final concentration of 0.5% and incubated at 95 °C for 10 min. Filter-aided sample preparation (FASP) trypsin digestion was performed as described previously [27] at a trypsin-to-substrate ratio of 1:50. The resulting peptides were acidified with formic acid (1% final concentration), desalted using C18 100 μL tips (Thermo Scientific) according to the manufacturer’s instructions, dried by vacuum centrifugation, and reconstituted in 20 μL of 0.1% formic acid before mass spectrometry. Per sample, 100 ng of peptides were analyzed using a nanoElute^®^ HPLC coupled to a TimsTOF Pro instrument (Bruker, Bremen, Germany). Peptides were separated on a reversed-phase analytical column (10 cm × 75 μm i.d., Bruker 1866154), and separated by running a gradient of 2 to 95% mobile phase B over 65 min (2% to 4% solvent B (0 to 1 min), 4 to 20% solvent B (1 to 46 min), 20 to 32% solvent B (46 to 60 min), 32 to 95% solvent B (60 to 61 min), and 95% solvent B (61 to 65 min) at a constant flow rate of 250 nL/min. The column temperature was maintained at 40 °C. MS analysis of eluting peptides was performed in ddaPASEF mode (1.1-s cycle time) as recommended by the manufacturer.

Mass spectrometry raw files were processed with MaxQuant (v.2.4.2.0) [28]. A peptide search was performed against an ENSEMBL [29] *Sus scrofa* proteome database (v.11.1.2021-11-10) and an NCBI ASFV Georgia (v.FR682468.2) proteome database. The ASFV Georgia proteome was used because it shares 99.99% genome sequence identity with the Armenia/07 isolate, which lacks a publicly available, reviewed proteome. The false discovery rate (FDR) was set to 0.1%, two missed cleavages were tolerated, the carbamidomethylation of cysteine residues was set as fixed modification, and protein N-terminal acetylation and the oxidation of methionine as variable modifications. The results from Maxquant were further analyzed using Perseus software (v.2.0.3.0) [30]. A protein was considered as identified if at least two unique peptides were found in three out of four of the replicates. Proteins specifically binding to VPS39 bait were filtered out by removing the GFP background. The background list consists of proteins identified in our GFP negative controls. Potential interactors were considered specific if they were identified only in GFP-bait pulldowns or if the log2 fold change (between GFP control and GFP-bait) was greater than 2 and the *p*-value of a two-sided *t*-test was <0.01.

## 3. Results

### 3.1. VPS39 Is a Common Interactor of ASFV Proteins CP204L and A137R

**Figure 1:**
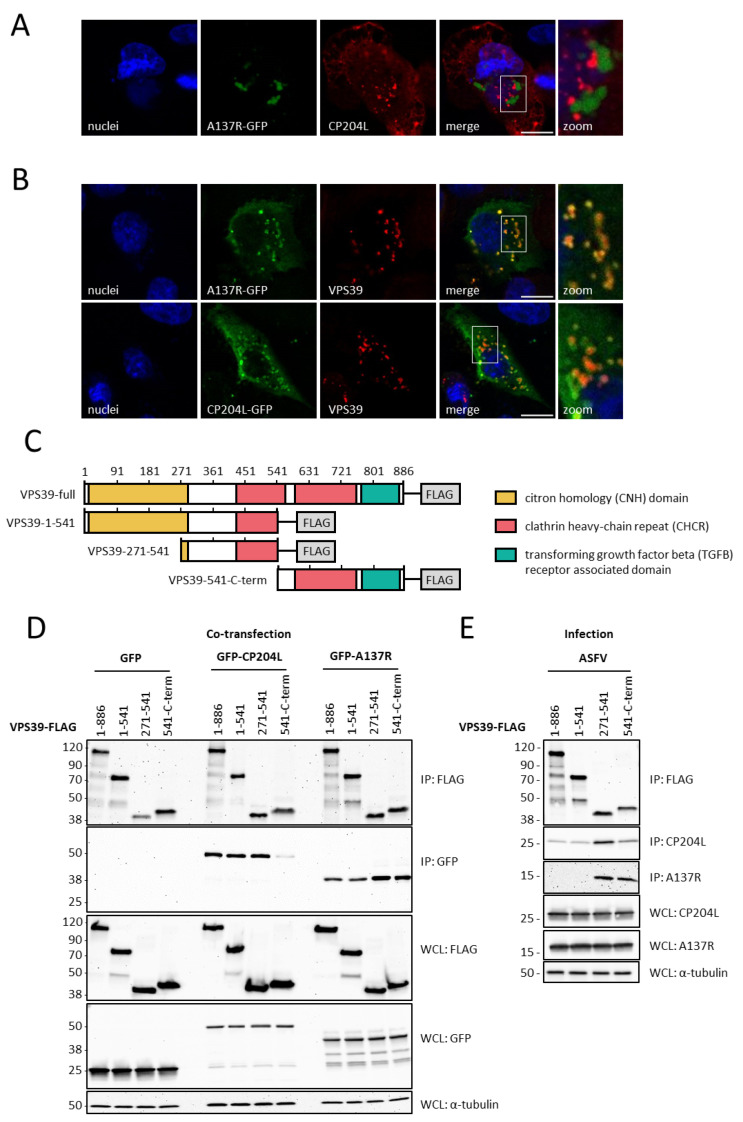
The interaction of ASFV proteins CP204L and A137R with the host protein VPS39. (**A**) Subcellular localization of GFP-tagged A137R and endogenous CP204L in WSL cells 24 h after ASFV infection. (**B**) Colocalization of CP204L-GFP and A137R-GFP with VPS39 in WSL cells. Scale bars, 10 µm. (**C**) Diagram of the different truncation FLAG-tagged fragments and the predicted domain organization of porcine VPS39 protein. The co-immunoprecipitation of FLAG-tagged VPS39 fragments with CP204L and A137R in WSL cells (**D**) 24 h after GFP-CP204L or A137R-GFP plasmid transfection and (**E**) 24 h after ASFV infection. Representative immunoblots of whole-cell lysates (WCLs) and GFP immunoprecipitates (IPs) are shown. α-tubulin was used as a loading control in WCLs.

A previous study reported co-immunoprecipitation between ASFV proteins CP204L and A137R [7]. Yet, reverse pulldown experiments failed to detect a direct interaction between these two proteins. Here, using immunofluorescence microscopy on infected WSL cells, we confirmed that A137R and CP204L do not directly colocalize as their fluorescence signals do not overlap. However, both proteins do aggregate in close vicinity, suggesting a potential indirect interaction mediated by a shared protein partner (Figure 1A). Therefore, we decided to examine whether A137R could interact with VPS39, a known interactor of the ASFV CP204L protein and a component of the endocytic pathway. We transfected WSL cells with A137R-GFP or CP204L-GFP expression vectors and visualized GFP fluorescence along with the endogenous levels of VPS39 using fluorescence microscopy. The images revealed colocalization between VPS39 and both ASFV proteins (Figure 1B). To identify a VPS39 domain involved in the interaction with CP204L and A137R, three constructs expressing individual VPS39 domains fused to the Flag were designed. The domains were specified as defined by Caplan et al., 2001 [31] and according to the InterPro [32] prediction (Figure 1C). We first co-expressed VPS39 protein fragments together with CP204L or A137R GFP constructs for 24 h in WSL cells. Subsequently, immuno-precipitated proteins were subjected to Western blot detection using an anti-GFP antibody (Figure 1D). The results confirmed that CP204L interacts with a clathrin heavy-chain repeat (CHCR) located between amino acids 271 and 541 of VPS39. A137R demonstrated an interaction with the same domain, as well as with a CHCR fragment situated between amino acids 541 and 886. Next, we conducted the immunoprecipitation of VPS39 fragments within ASFV-infected WSL cells, using antibodies against CP204L and A137R for viral protein detection. As shown in Figure 1E, both viral proteins were found to interact with the VPS39^271–541^ fragment. However, only A137R exhibited a strong signal indicating interaction with the VPS39^541-C-term^ fragment. Interestingly, endogenous A137R did not interact with a full-length VPS39 or the VPS39^1–541^ fragment.

### 3.2. Functional Profiling of Proteins Associated with VPS39

To further characterize the host protein interactome of VPS39 in ASFV-infected cells, we utilized an affinity tag purification–mass spectrometry (AP-MS) approach, following the previously described procedures [7]. WSL cells stably expressing porcine VPS39 tagged with a C-terminal GFP as bait were used together with GFP-expressing WSL cells serving as negative controls. This analysis identified 809 proteins (Supplementary Table S1) that specifically co-immunoprecipitated with VPS39. Among these, CP204L and A137R were the only viral proteins that specifically interacted with VPS39. Then, we analyzed the functional enrichment of all identified PPI partners using the R package “clusterProfiler”. The Gene Ontology (GO) term enrichment analysis included biological process (BP) and cellular component (CC) categories. The most significant results of the GO enrichment analysis ranked by *p*-value were provided in Table 1. The enrichment results for the BP GO category showed that the VPS39-associated proteins were primarily involved in ribose 5-phosphate and ATP metabolic processes (GO:0019693), cellular respiration (GO:0015980), vesicle-mediated (GO:0016192), and nuclear transport (GO:0051169), DNA replication (GO:0006260), and protein–RNA complex assembly (GO:0071826). For the CC GO category, the results indicated that these interacting proteins are primarily localized in mitochondria (GO:0005739), various functional protein complexes, and associated with membranes.

### 3.3. Mitochondrial Protein Interactome of VPS39

**Figure 2:**
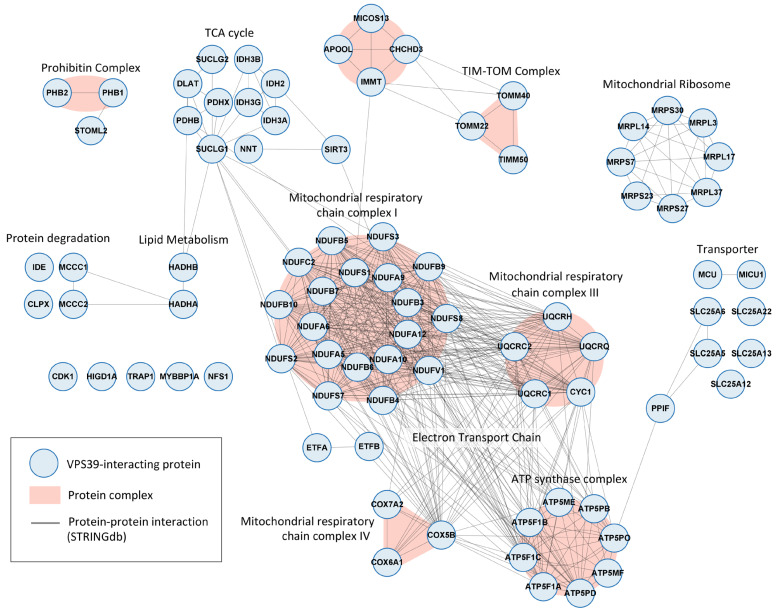
Network illustrating the interactions between VPS39-associated proteins localized in mitochondria. Proteins were grouped into protein complexes according to the EBI Complex Portal and visualized with STRING to illustrate the experimentally validated PPIs among them.

The most significantly enriched group of mitochondrial proteins copurifying with VPS39 was illustrated in an interaction subnetwork (Figure 2). We used the STRING database [33] to identify the interactions between host proteins that have been experimentally validated and the EBI Complex Portal [34] database to assign the proteins to specific protein complexes. In addition to the mitochondrial protein complexes involved in aerobic respiration, we observed that VPS39 associates with the components of other protein complexes, including the mitochondrial contact site and cristae organizing system (MICOS) complex, the translocases of the inner and outer membrane (TIM-TOM) complex, and the prohibitin complex. The top three ranked high-confidence VPS39 interactors, based on their abundance, sequence coverage, and probability score, were ATP synthase subunits alpha (ATP5F1A) and beta (ATP5F1B) and the translocase of the outer mitochondrial membrane 22 (TOMM22) (Table 2).

## 4. Discussion

Viruses modulate the biology of infected cells by forming extensive interfaces with host proteins. For this purpose, many viruses encode multifunctional proteins, which can interact with multiple cellular partners to regulate diverse cellular functions. Several ASFV proteins have been reported to exhibit multifunctionality and also target common cellular components [6]. While most ASFV–host interaction studies focus on proteins linked to innate immunity, identifying the cellular targets involved in diverse biological processes is crucial for a comprehensive understanding of ASFV pathogenicity. High-throughput techniques, such as AP-MS, provide a global and unbiased approach to identifying virus–host protein interactions and have already been successfully applied in some ASFV studies [11,35,36]. Additionally, computational proteome-wide PPI screening tools, like AlphaFold, have recently proven useful in predicting interactions between ASFV proteins [37].

This study uncovers VPS39 as a common cellular target for ASFV proteins CP204L and A137R. Both viral proteins interact with VPS39 via its CHCR domains. Interestingly, A137R can interact with both CHCR domains spanning amino acids 271–541 and 541–886, whereas CP204L’s interaction is notably enhanced by the presence of the CHCR domain between amino acids 271 and 541. The CHCRs identified in non-clathrin proteins are likely to facilitate protein–protein interactions or perform clathrin-like functions. In VPS39 specifically, the CHCRs were reported to be critical for their association with endosomal membranes and protein homo-oligomerization [38]. The earlier study established that CP204L’s interaction with VPS39 disrupts its binding to endolysosomal proteins, causing their aggregation at the virus replication site [7]. Endosomal compartments may provide a scaffold for virus replication and function as intermediates for virus assembly. However, the exact function of endosomal components in ASFV replication has yet to be elucidated.

Our analysis of the VPS39 interactome in ASFV-infected cells suggests that the function of VPS39 extends beyond the process of endosomal membrane redistribution. VPS39 associates with numerous proteins implicated in diverse cellular processes, with notable enrichment observed in mitochondrial proteins. Interestingly, VPS39 interacts with the TOM mitochondrial complex, facilitating the formation of dynamic contact sites between mitochondria and lysosomes [18,39]. In addition to VPS39 interactions with TOM complex components TOMM22 and TOMM40, we identified interactions with the MICOS complex, a critical regulator of cristae architecture and electron transport chain (ETC) function [40]. Recently, the human cytomegalovirus was shown to target the MICOS complex during infection to increase mitochondrial bioenergetics [41]. Beyond their essential role in energy supply during replication, mitochondria also play a pivotal role in inducing immune responses [42]. Although it is known that mitochondria are recruited to ASFV factories and actively supply ATP for ASFV replication and assembly [43,44], the mechanisms underlying this process remain unknown. Here, we showed that VPS39 could be an important hub mediating virus–mitochondria interaction and a potential therapeutic target for limiting essential energy resources required for viral replication. However, further research is required to analyze the role of VPS39 in regulating mitochondrial bioenergetics and innate immune signaling.

Our analysis of VPS39 interactions in ASFV-infected cells identified A137R and CP204L as specific interactors of VPS39. No additional viral proteins were found to specifically interact with VPS39 according to our analysis criteria. However, we cannot strictly exclude interactions between VPS39 and other viral proteins. Specifically, interactions that are physically instable, only transiently established during specific periods of the infection cycle, do not occur in WSL cells, or are not compatible with the used affinity tag may have escaped detection.

During infection, both viral proteins A137R and CP204L engage with a CHCR domain spanning amino acids 271–541, indicating their competitive interactions for binding to VPS39 with cellular partners and among themselves. CP204L predominantly interacts with VPS39 early in infection [7], whereas A137R is expressed later during infection [10]. This distinction suggests a potential temporal separation between the interactions of VPS39 with these two viral proteins. Considering the currently known properties of viral proteins A137R and CP204L, we propose that VPS39 may serve two distinct functions.

First, the direct binding of CP204L to VPS39 may protect the viral protein from Ras-related protein Rab-1b (RAB1B)-mediated autophagic degradation. Recently, Yang et al. [12] showed that sorting nexin 32 (SNX32) and RAB1B recruited CP204L into the protein complex, targeting it for autophagic degradation. VPS39 may prevent CP204L degradation through two mechanisms: directly, by competing for the same binding site on CP204L as SNX32 and RAB1B; or indirectly, by disrupting the fusion of autophagosomes with lysosomes—thus inhibiting CP204L autophagy. Although ASFV already employs multiple mechanisms to inhibit autophagy early in infection [45], the binding of VPS39 to CP204L could provide immediate and specific additional protection to ensure its localization to the newly formed viral replication sites before further viral mechanisms are implemented to control protein degradation.

Second, the interaction between A137R and VPS39 could play a role in counteracting immune responses during ASFV infection. Sun et al. [13] discovered that A137R inhibits type I interferon production by interacting with TANK-binding kinase 1 (TBK1) and promoting its lysosomal degradation through an unknown mechanism. Additionally, A137R inhibits MAPK and NF-κB signaling by suppressing myeloid differentiation primary response protein (MyD88) [46]. We hypothesize that A137R could facilitate the degradation of immune-related proteins by binding to the CHCR domain spanning amino acids 541–886 of VPS39. Importantly, this interaction would not hinder VPS39 from integrating into the HOPS complex via its CHCR domain (amino acids 271–541), which binds to a VPS11 component. Therefore, immune proteins associated with A137R and VPS39 would be directed toward lysosomes or autophagosomes for degradation.

Collectively, our analysis of VPS39′s protein interactions indicates its potential to modulate mitochondrial dynamics by forming membrane contact sites with other organelles, possibly through interactions with TOM complex proteins. Furthermore, its association with the MICOS complex could enhance mitochondrial energy production, promoting ASFV replication. Further experiments are necessary to confirm and clarify how VPS39 interacts with mitochondria and explore its potential role in preventing CP204L degradation and supporting the degradation of immune proteins by A137R.

## Figures and Tables

**Table 1:** Top gene ontology terms enriched among host proteins associated with VPS39.

GO ID	GO Term Description	Count	*p*-Value
**Biological process**			
GO:0046390	ribose phosphate biosynthetic process	56	5.45 × 10^−25^
GO:0045333	cellular respiration	57	5.45 × 10^−25^
GO:0006754	ATP biosynthetic process	28	3.20 × 10^−18^
GO:0016192	vesicle-mediated transport	47	1.87 × 10^−12^
GO:0071826	protein-containing complex assembly	35	3.59 × 10^−9^
GO:0006260	DNA replication	40	1.44 × 10^−9^
GO:0051169	nuclear transport	37	3.84 × 10^−7^
**Cellular component**			
GO:0005739	mitochondrion	71	9.10 × 10^−34^
GO:1902494	catalytic complex	35	1.64 × 10^−17^
GO:0070469	respirasome	29	2.01 × 10^−15^
GO:0016020	membrane	27	7.16 × 10^−15^
GO:1990904	ribonucleoprotein complex	19	3.08 × 10^−8^

GO ID refers to the gene ontology identification number. The count is the number of genes in the query gene list. The *p*-value represents the statistical significance of the gene enrichment test.

**Table 2:** Top 3 high-confidence mitochondrial proteins identified as interactors for VPS39 in AP-MS experiments.

Gene	Protein Name	Protein Score	Seq. Coverage	Abundance (log10 iBAQ)
ATP5F1B	ATP synthase subunit beta	323.31	71.1	5.30
ATP5F1A	ATP synthase subunit alpha	323.31	57.7	5.36
TOMM22	translocase of outer mitochondrial membrane 22	317.52	49.6	5.42

## Data Availability

All mass spectrometry raw data and MaxQuant output tables were deposited in the ProteomeXchange Consortium (http://proteomecentral.proteomexchange.org, accessed on 24 July 2024) via the PRIDE partner repository [47] and is publicly available under identifier PXD054169.

## Supplementary Materials

The following supporting information can be downloaded at: https://www.mdpi.com/article/10.3390/v16091478/s1, Table S1: Collection of proteomics data including: List of background proteins (Table S1A); List of proteins identified as VPS39 interactors in AP-MS experiment (Table S1B); Results of the GO term enrichment analysis (Table S1C); List of mitochondrial protein complexes interacting with VPS39 (Table S1D).
